# Structure, dynamics and predicted functional role of the gut microbiota of the blue (*Haliotis fulgens*) and yellow (*H. corrugata*) abalone from Baja California Sur, Mexico

**DOI:** 10.7717/peerj.5830

**Published:** 2018-11-02

**Authors:** Francesco Cicala, José Alejandro Cisterna-Céliz, James D. Moore, Axayácatl Rocha-Olivares

**Affiliations:** 1 Molecular Ecology Laboratory, Department of Biological Oceanography, CICESE, Ensenada, Baja California, Mexico; 2 Bodega Marine Laboratory, University of California, Davis, Bodega Bay, CA, United States of America

**Keywords:** *Haliotis fulgens* and *H. corrugata* microbiota composition, Bacterial interaction, Ecological function predictions., 454 Pyrosequencing

## Abstract

The GI microbiota of abalone contains a highly complex bacterial assemblage playing an essential role in the overall health of these gastropods. The gut bacterial communities of abalone species characterized so far reveal considerable interspecific variability, likely resulting from bacterial interactions and constrained by the ecology of their abalone host species; however, they remain poorly investigated. Additionally, the extent to which structural changes in the microbiota entail functional shifts in metabolic pathways of bacterial communities remains unexplored. In order to address these questions, we characterized the gut microbiota of the northeast Pacific blue (*Haliotis fulgens* or HF) and yellow (*Haliotis corrugata* or HC) abalone by *16S rRNA* gene pyrosequencing to shed light on: (i) their gut microbiota structure; (ii) how bacteria may interact among them; and (iii) predicted shifts in bacterial metabolic functions associated with the observed structural changes. Our findings revealed that* Mycoplasma* dominated the GI microbiome in both species. However, the structure of the bacterial communities differed significantly in spite of considerable intraspecific variation. This resulted from changes in predominant species composition in each GI microbiota, suggesting host-specific adaptation of bacterial lineages to these sympatric abalone. We hypothesize that the presence of exclusive OTUs in each microbiota may relate to host-specific differences in competitive pressure. Significant differences in bacterial diversity were found between species for the explored metabolic pathways despite their functional overlap. A more diverse array of bacteria contributed to each function in HC, whereas a single or much fewer OTUs were generally observed in HF. The structural and functional analyses allowed us to describe a significant taxonomic split and functional overlap between the microbiota of HF and HC abalone.

## Background

The gastro-intestinal tract (or GI) of metazoans may be considered a highly complex ecosystem inhabited by a large number of bacteria (Backhed, 2005). For instance, the commensal microbiota harbored by the human GI far exceeds the total number of cells in the entire human body, and their collective genome (microbiome) is orders of magnitude larger than our own (Backhed, 2005; Bates et al., 2006). Moreover, the GI microbiome has been associated with essential physiological activities such as food digestion, nutrient assimilation, and defense against invasion of foreign bacterial species; which in turn may prevent epidemiologic outbreaks (Ten Doeschate & Coyne, 2008; Zhao et al., 2012; Blaut & Clavel, 2007). Also, functional studies have revealed that the relationship between the gut microbiome and its host may be so close that bacteria may be directly involved in the maturation of the GI tract of the hosts species (Bates et al., 2006; Bry et al., 1996; Bano et al., 2007).

Abalone (or *Haliotis* spp.) are worldwide gastropods that play essential ecological roles. Adult abalone are considered ecosystem engineers, as they graze macro and micro-algae at species-specific depths, which maintains open habitat exploited by other benthic organisms (Crosson & Friedman, 2017; Cox, 1962). Several abalone species support valuable fisheries and aquaculture production in many countries around the world (Crosson & Friedman, 2017). For example, the peninsula of Baja California harbors seven exploitable abalone species (Morales-Bojórquez, Muciño Díaz & Vélez-Barajas, 2008), two of which, the blue abalone *Haliotis fulgens* (HF, henceforth) and the yellow abalone *Haliotis corrugata* (HC, henceforth), sustain the high-valued fishery in the NW Mexican Pacific coast (Morales-Bojórquez, Muciño Díaz & Vélez-Barajas, 2008; SAGARPA, 2009).

As documented by cultured and uncultured approaches, the composition of the abalone gut microbiota may be influenced by a variety of factors such as diet, environmental conditions and ontogenetic stages (Ten Doeschate & Coyne, 2008; Zhao et al., 2012; Meryandini, Junior & Rusmana, 2015; Sawabe et al., 2003; Tanaka et al., 2003; Pang, Xiao & Bao, 2006). Also, the use of probiotics has revealed that interspecific bacterial relationships may shape the final gut microbiome composition of several marine invertebrates, including abalone (Meryandini, Junior & Rusmana, 2015; Sawabe et al., 2003; Tanaka et al., 2003). Overall, these factors may explain the consistent differences in the gut microbiome of abalone species studied so far. In this context, the most abundant bacteria in homogenate samples of the entire GI of *H. discus hannai* were fermenter *Gammaproteobacteria*, such as *Vibrio halioticoli* as well as other *Vibrio* species, *Alphaproteobacteria*, *Mollicutes* and *Fusobacteria* (Tanaka et al., 2003; Tanaka et al., 2004). Moreover, the intestinal microbiota (from stomach to anus) of *H. diversicolor* was dominated by *Mollicutes*, *Flammeovirga*, as well as *Alpha-, Beta-*, *Gamma*- and *Delta- proteobacteria* (Huang et al., 2010). In contrast, the bacterial composition of *H. gigantea* (from homogenate samples of the entire GI) appears less complex with a preponderance of *Gammaproteobacteria* and *Mollicutes* (Iehata et al., 2014).

Despite the importance of the GI microbiomes for the survival of blue and yellow abalone, no efforts have been made to characterize them. Furthermore, it is equally uncertain which factors may shape their final composition as well as the functional roles played by the most representative bacterial groups. Accordingly, we hypothesize that the composition of GI microbiomes harbored by HF and HC abalone may reflect species-specific ecological adaptations; for example, they may change in response to the distinct bathymetric distribution of these species (3–4 m in the shallow HF and as deep as 25 m for HC). To analyze the structure and variability of the GI microbiota of wild-caught specimens of HC and HF, we collected post esophageal tissues of both abalone species and sequenced *16S rRNA* gene amplicons using 454 pyrosequencing (Roche). Profiling of detected OTUs was used to characterize bacterial communities according to qualitative (e.g., presence/absence) and quantitative (e.g., read abundance) analyses. Additionally, the phylogenetic distance among microbial communities was assessed using unweighted UniFrac metrics. Finally, we analyzed the functional consequences of structural changes using a predictive metagenomic analysis.

## Materials and Methods

### Sample collection and genetic analyses

Wild abalone (*n* = 31 HF, *n* = 35 HC) were collected from the commercial harvest along the Pacific coast Baja California (Mexico) from Isla Natividad (27°53′33″N 115° 13′19″W) to Punta Abreojos (26°42′52″N 113°34′20″W), during two field expeditions organized in April and November 2012 (Table S1). In the field, approximately 30 mg of post esophageal tissue were dissected from visually healthy animals bearing no signs of the withering syndrome (Friedman, 2012), and immediately transferred to sterile 1.5 ml microcentrifuge tubes containing molecular grade ethanol, until further analysis. Total DNA was extracted and purified from preserved tissues using DNeasy blood & tissue kit (Qiagen, Valencia, CA, USA) following manufacturer’s protocols.

A fragment of the bacterial ribosomal *16S rRNA* spanning V1–V3 regions was PCR amplified using universal eubacterial primers 28F: 5′-GAGTTTGATCNTGGCTCAG- 3′ (Ludwig, Mittenhuber & Friedrich, 1993) and 519R: 5′-GTNTTACNGCGGCKGCTG - 3′ (Ruff-Roberts, Kuenen & Ward, 1994). PCR reactions (20 µl) contained: 1X PCR Buffer (Kapa Biosystems, Woburn, MA, USA), 1.5 mM magnesium chloride (Kapa Biosystems, Woburn, MA, USA), 0.2 mM dNTPs (New England Biolabs, Beverly, MA, USA), 0.5 µM each primer, 0.4 mM bovine serum albumin (New England Biolabs, Beverly, MA, USA), 1U *Taq* polymerase (Kapa Biosystems, Woburn, MA, USA), and 100 ng purified DNA. Thermal cycling consisted of an initial incubation at 94 °C for 4 min, followed by 40 cycles of: 94 °C for 1 min; 62 °C for 30 s and 72 °C for 30 s, and a final incubation of 8 min at 72 °C. Confirmation of amplification was carried out by 1.5% agarose gel electrophoresis. Amplicons were subsequently tagged using Roche 454 adaptors and multiplex identifier (MID) tags for each organism, following the bacterial tag-encoded FLX amplicon pyrosequencing (bTEFAP) approach of Dowd et al. (2008). Following normalization, Roche 454 pyrosequencing was carried out in a GS FLX Titanium platform by Research and Testing Laboratory (Lubbock, TX, USA).

Finally, given that different DNA extraction and/or purification methods may introduce bias against either gram positive or negative bacteria in purified DNA (Yuan et al., 2012; Gill et al., 2016), we evaluated the introduction of such bias in our samples. We tested in a random subset of eight DNA samples (four from each species) their suitability for amplifying gram negative and gram positive DNA using gram-specific forward primers (16S_68d for gram negative and 16S_143 for gram positive) (Klausegger et al., 1999). PCR reactions (15 µl) contained: 1×  PCR Buffer (Kapa Biosystems, Woburn, MA, USA), 1 mM magnesium chloride (Kapa Biosystems, Woburn, MA, USA), 0.4 mM dNTPs (New England Biolabs), 0.4 µM each primer, 1U *Taq* polymerase (Kapa Biosystems, Woburn, MA, USA), and 90 ng purified DNA. The same thermal cycling conditions were used as described for universal eubacterial primers, except that the annealing temperatures were 55 and 60 °C for gram positive and negative specific primers, respectively. Confirmation of amplification was carried out by 1.5% agarose gel electrophoresis.

### Bioinformatic analyses

The *16S rRNA* reads were analyzed using Quantitative Insights Into Microbial Ecology (QIIME) software version 1.9.1 (Caporaso et al., 2010b). The first step consisted in demultiplexing; subsequently, reads were filtered according to Phred quality scores obtained from the 454 pyrosequencing. Acceptance quality criteria consisted of: (i) minimum and maximum lengths of 250 and 550 bp, respectively; (ii) default minimum quality Phred score of 25; (iii) maximum homopolymer length of 6 bp.

Sequences that met quality criteria were initially filtered using USEARCH (Edgar, 2010) to detect and remove chimeras as implemented in QIIME pipeline (Caporaso et al., 2010b) and then clustered in operational taxonomic units (OTUs) at 97% sequence similarity using the UCLUST algorithm (Edgar, 2010). Taxonomic assignment was carried out using the longest sequence from each OTU and SILVA 128 database (Released: 28.09.2016 containing 646,151 sequences; http://www.arb-silva.de/). OTUs with total read number <20 were discarded; remaining OTUs were then aligned using Python Nearest Alignment Space Termination (PyNAST) algorithm (Caporaso et al., 2010a). The alignment was filtered to remove gaps and outliers (e.g., sequences dissimilar to the alignment consensus) using default QIIME parameters.

Additionally, to evaluate the potential bacterial origin of DNA of Unassigned OTUs (with no match in SILVA database), we aligned them with bona fide representative *16S rDNA* sequences of Eubacteria, chloroplasts and Archean species from GenBank and reconstructed a Neighbor-joining (NJ) tree using MEGA 6 (Tamura et al., 2013). OTUs that fell outside of the Eubacterial lineage were removed from the PyNAST alignment before further analyses.

The number of reads of each abalone was normalized using the rarefication method (Weiss et al., 2017). Normalization was achieved by randomly subsampling 500 reads per abalone; we selected a sample size as close as possible to the asymptotic plateau of rarefaction curves in most abalone (Fig. S1).

Principal coordinate analysis (PCoA) based on unweighted unique fraction metric (or UniFrac) distance was performed to compare the microbiota among HF and HC abalone species from an ecological-phylogenetic framework. Results were visualized using EMPeror (Vázquez-Baeza et al., 2013). Briefly, unweighted UniFrac is a qualitative β diversity analysis measuring the distance between two or more communities, as the fraction of the branch length in a phylogenetic tree that leads to descendants from either one community, but not both (Lozupone et al., 2007; Lozupone & Knight, 2005). Accordingly, this measure may reflect essential microbial adaptations to one environment (Lozupone & Knight, 2005). Statistical evaluation of UniFrac results was carried out using ANOSIM as implemented in QIIME (Caporaso et al., 2010b).

### Ecological analysis

The normalized number of reads was used as abundance proxy to estimate diversity and structure of the abalone gut microbiota. Differences in the bacterial composition between abalone species were evaluated by a linear discriminant analysis (LDA) effect size (LEfSe) (Segata et al., 2011).

**Figure 1:**
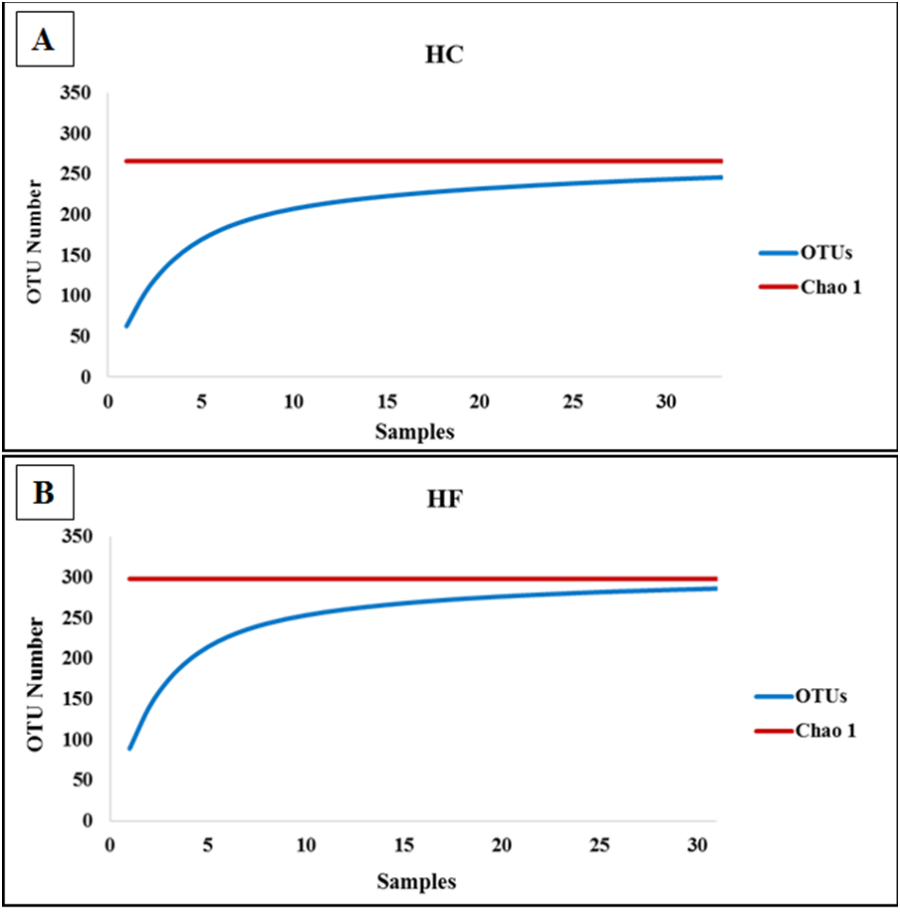
Diversity as function of sample size. Rarefaction curves of the cumulative number of observed OTUs with increasing number of samples (individual abalone) and Chao 1 estimation of total OTU richness in microbiomes of (A) *H. corrugata* (HC) and (B) *Haliotis fulgens* (HF).

To assess how exhaustively bacterial communities of both abalone species were sampled, rarefaction curves of discovered OTUs were generated for increasing numbers of sampled abalone (Fig. 1). Also, OTU abundance was used to compute the non-parametric species richness estimator Chao 1 (Chao, 1984). Rarefaction curves were obtained using the EstimateS V.9.0.1 program (Colwell, 2013).

Microbiome community structure was evaluated using non-parametric multidimensional scaling (MDS) analyses using Bray–Curtis and Sorensen similarity indices based on read abundance and on presence/absence, respectively, as implemented in PRIMER V.6 (Clarke & Warwick, 2001). The statistical comparison of MDS results was performed with ANOSIM as implemented in Past V. 2.17c (Hammer, Harper & Ryan, 2001) using OTUs assigned to bacterial taxa matching SILVA database. To determine which OTUs were primarily responsible for the dissimilarity between HF and HC, a SIMPER analysis (Table S2) with square root transformed data was performed using PRIMER V.6 (Clarke & Warwick, 2001). In order to increase the number of OTUs used in the characterization of microbiomes, we repeated the analyses also including SILVA unassigned OTUs that were phylogenetically identified as bacteria, as described above.

Bacterial interactions in the microbiomes of both abalone species were estimated using Jaccard distance (*J*_*d*_) as implemented in PRIMER V.6 (Clarke & Warwick, 2001). *J*_*d*_ is a measure of dissimilarity for all pairwise combinations of a data set (Dowd et al., 2008) and was calculated using OTUs presence/absence. *J*_*d*_ values close to 0 (from 0 to 0.33) were interpreted as co-occurrence (or putative mutualistic relationships) and values close to 1 (from 0.68 to 1) as interactions leading to exclusion (or putative competitive) (Rahel, 2000); whereas intermediate values were considered as neutral relationships.

### Functional prediction of predominant bacterial species

A phylogenetic investigation of communities by reconstruction of unobserved states (PICRUSt) (Langille et al., 2013) was carried out to predict the functional attributes of metabolic genes from HF and HC GI microbiotas. Briefly, PICRUSt is a bioinformatic approach that uses information from a genetic marker, such as the *16S rRNA* gene, to predict the functional content of a bacterial community characterized by metabarcoding (Langille et al., 2013; De Voogd et al., 2015). Functional predictions are obtained by matching *16S rRNA* gene sequences against a genomic KEGG database, previous normalization of read numbers (Langille et al., 2013; De Voogd et al., 2015). The central result of PICRUSt consists of a table of the functional gene frequencies known as KEGG Orthologs (or KOs). KOs are hierarchically organized in sets of homologous sequences with known function and assigned to biological pathways. We analyzed the data using the raw KOs counts as well as categorizing them by biological pathway. PICRUSt analyses use Greengenes (last released in 2013 containing 202,421 bacterial and archaeal sequences, http://greengenes.lbl.gov) as taxonomic and functional reference database. To implement quality control, we computed weighted nearest sequenced taxon index (NSTI) values for the metabarcodes of each abalone. NSTI was developed to evaluate the prediction accuracy of PICRUSt, since it reflects the average genetic distance (measured as number of substitutions per site) between an OTU against a reference genome (Langille et al., 2013; De Voogd et al., 2015). Following suggested guidelines (Langille et al., 2013), we eliminated observations with a NSTI higher than 0.17.

In order to assess the contribution of individual OTUs to predicted KO functions, first we focused our analyses on genes involved in metabolic pathways (KEGG IDs from EC:1.1.1 to EC:6.5.1). Next, we categorized the relative importance of KO genes by ranking them according to their counts. These counts were obtained with PICRUSt v.1.1.0 (Langille et al., 2013) and were log transformed to rank their relative abundance (Urbanová & Bárta, 2014). We focused our attention on a chosen subset of KOs with the highest counts (*n* = 10 selected at random), as a first order analysis to characterize functional differences between these microbiotas. Significance of differences in the contribution of OTUs to these KOs was evaluated non-parametrically using a *χ*^2^ test.

Finally, to compare the functionality of individual microbiomes in both species, non-parametric MDS analyses based on Bray–Curtis similarity using fourth root transformation were carried out using the count number of identified KEGG orthologs and the count number of predicted metabolic functions in PRIMER V6 (Clarke & Warwick, 2001).

## Results

### Pyrosequencing and metabarcoding results

Pyrosequencing yielded 451,095 raw *16S rRNA* reads of which 239,125 met quality criteria and were non-chimeric, these were assigned to 1,508 OTUs. A number of these OTUs were removed from subsequent analyses, 1,066 because their read number was <20 and an additional 128 because they were genetically dissimilar from bona fide bacterial *16S rRNA* sequences. The remaining 314 OTUs (comprising 157,686 quality reads −66%-) included 58 Unassigned-OTUs (Table S3) whose phylogenetic position fell within the Eubacterial linage (Fig. S2), suggesting they originate from unknown or poorly characterized bacterial taxa.

The most diverse microbial phyla in the gut microbiota of both abalone species were (number and % of OTUs): *Tenericutes* (230, 73.2%), *Fusobacteria* (16, 5.1%), and *Proteobacteria* (9, 2.9%). The most abundant OTUs were assigned to class *Mollicutes* (order *Mycoplasmatales*). Notably, LEfSe analysis revealed that 130 of 230 *Mollicutes* OTUs were exclusive or predominant to either in HC or HF (Table S4). Similar results were observed for other predominant bacterial families such *Vibrionaceae* (Table S4).

The classes *Mollicutes*, *Fusobacteria, Alfa* and *Gamma-protobacteira* comprised 99% of the identifiable reads. Rarefaction curves suggest that the bacterial communities were sufficiently sampled in both abalone species, given their asymptotic shape and the proximity of the observed number of taxa found in each species to CHAO 1 estimates (Fig. 1). Normalization was achieved by standardizing to a subsampling depth of 500 randomly subsampled reads per abalone, which represents the number of reads close to the asymptote for most organisms (Fig. S1). As a result, five HC and two HF abalone with less than 500 reads were excluded from subsequent analyses.

**Figure 2:**
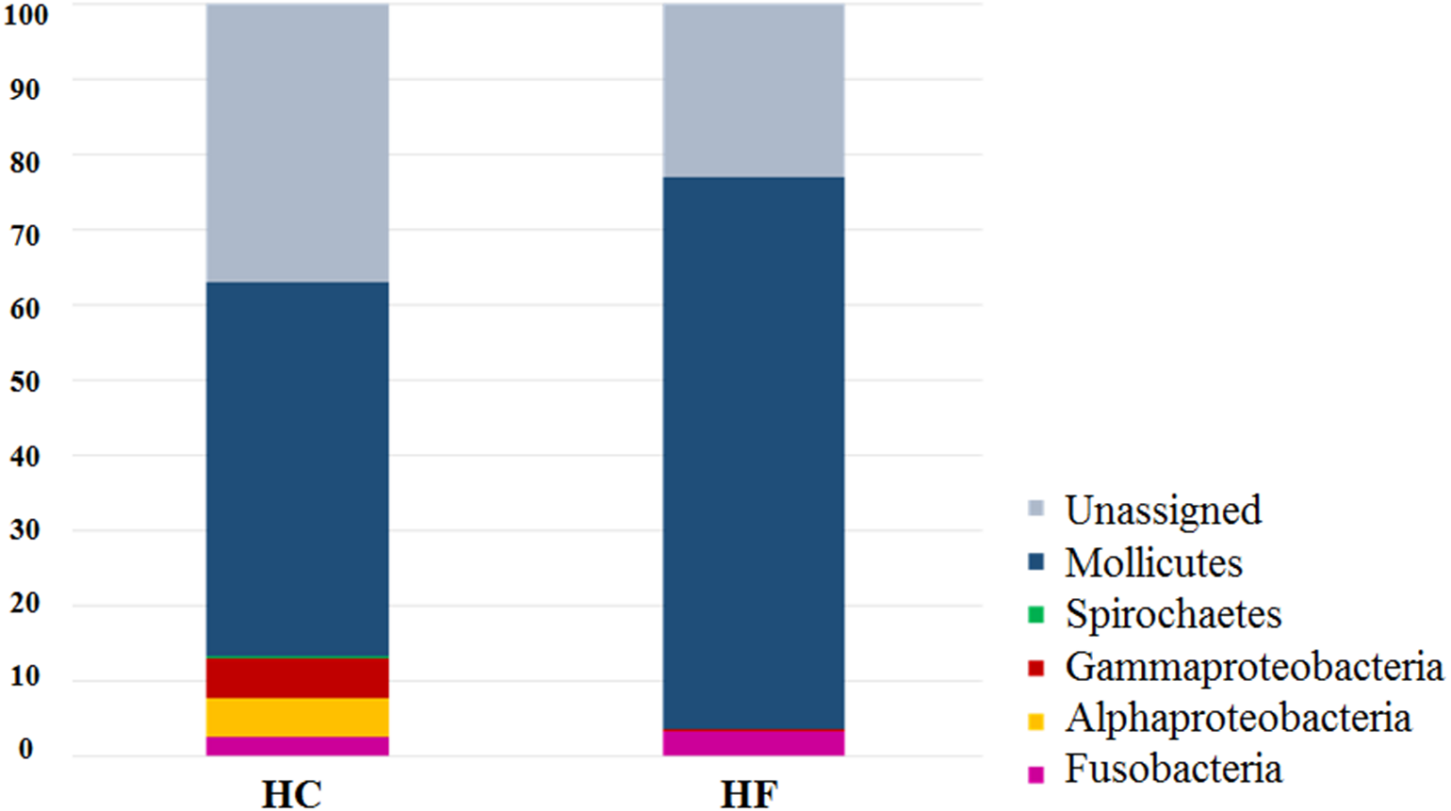
Species-specific GI tract microbiota composition. Major bacterial taxa comprising the gut microbiota of *H. corrugata* (HC) and *Haliotis fulgens* (HF).

**Figure 3:**
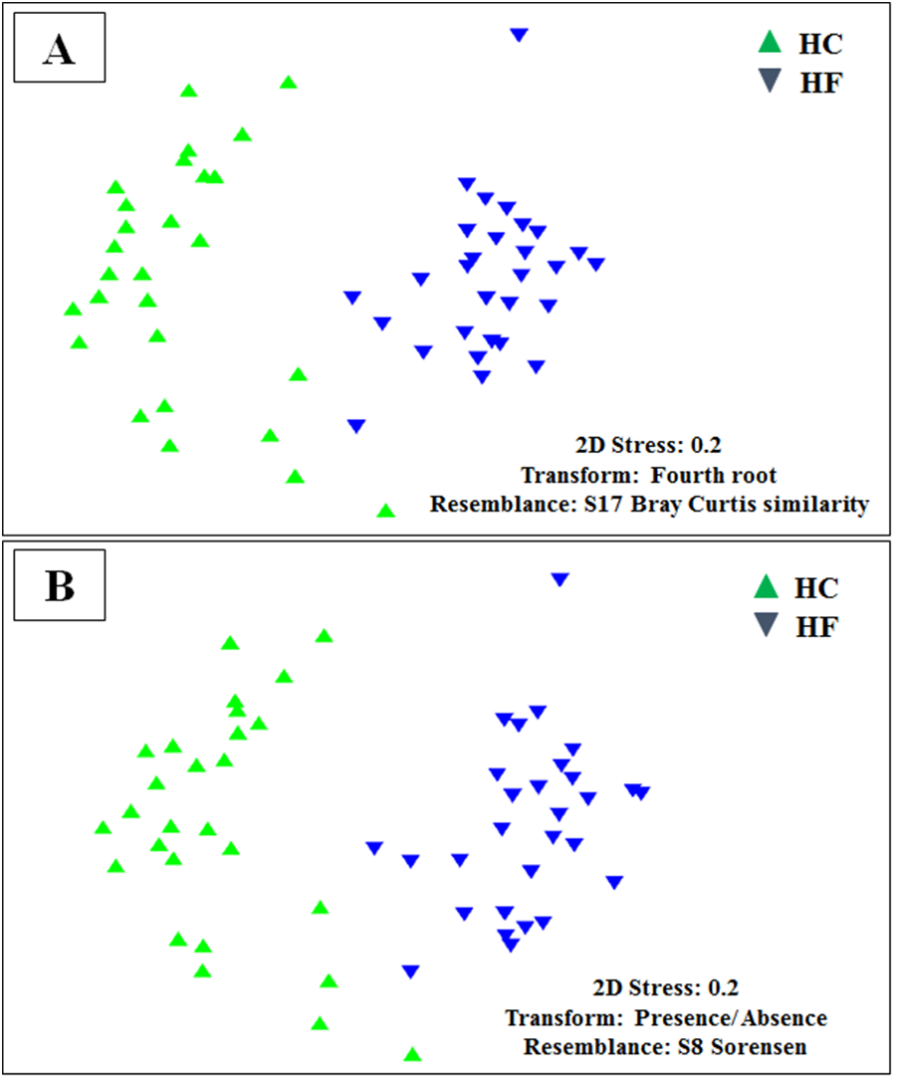
Microbiome structural variation. Non-metric multidimensional scaling (MDS) based on: (A) Bray Curtis similarity index using fourth root transformed read abundance and (B) Sorensen similarity index based on presence/absence. Both MDSs were obtained using assigned OTUs assembled at 97% similarity cut-off of the gut microbiota. HC: *Haliotis corrugata*, HF: *Haliotis fulgens*.

Despite the intra-specific compositional variation (Fig. S3) and similar community structure at the highest taxonomic resolution between abalone species (Fig. 2), MDS analyses performed using only bacterial OTUs, assigned using SILVA database, revealed a clear-cut structural difference between the GI bacterial metabarcodes of both species, both considering read number as a proxy of abundance and solely on the basis of presence/absence data (Fig. 3). Significant interspecific differentiation was corroborated by ANOSIM analyses in both cases (*R*_abundance_ = 0.786; *p* < 0.001; *R*_presence∕absence_ = 0.788; *p* < 0.001). Given the large fraction of unassigned OTUs (36%), the inclusion of originally unassigned OTUs but phylogenetically identified as bacteria produced similar results (Fig. S4; *R*_abundance_ = 0.792; *p* < 0.001; *R*_presence∕absence_ = 0.789; *p* < 0.001). This suggests that the distinction of both abalone microbiomes can be attributed to known and unknown bacterial taxa. PCoA based on unweighted UniFrac distance also supported the structural separation among HC and HF bacterial communities (Fig. S5; ANOSIM, *R*_unweighted UniFrac_ = 0.39; *p* < 0.001).

**Figure 4:**
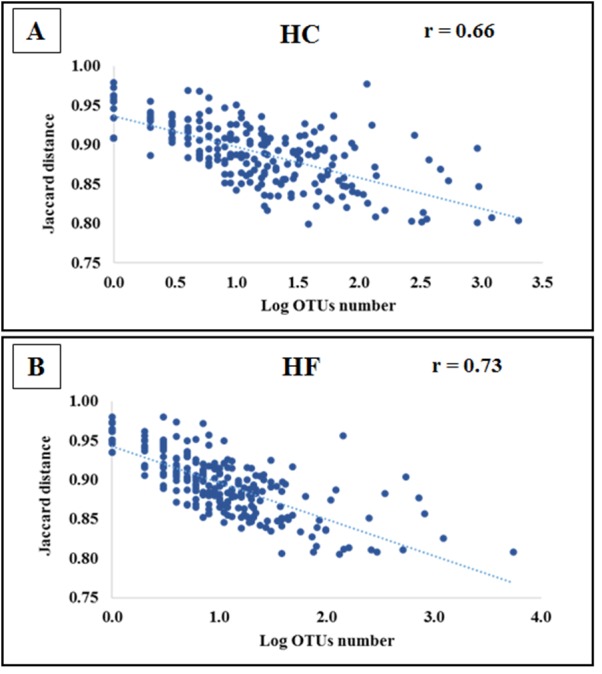
Bacterial species interactions as a function of their abundance. Correlation of average Jaccard distance based on all pairwise relationships as a function of the number of reads (log scale) in microbiomes of (A) *Haliotis corrugata* and (B) *Haliotis fulgens*.

Jaccard distances revealed that interspecific relationships among OTUs changed by an order magnitude with most involving competition (HC: 23,588 and HF: 30,129), followed by neutral (HC: 1851 and HF: 1879) and a smaller number of mutualistic interactions (HC: 212 and HF: 123). Furthermore, the ${\bar {J}}_{d}$ of the majority of OTUs decreased significantly with increasing read number in both species (*p* < 0.001; Fig. 4).

Our use of specific primers corroborated the presence of amplifiable DNA from gram-positive and gram-negative bacteria in the extracted DNA, hence we posit that the observed biased composition is not an artifact of the DNA extraction method (Fig. S6).

### Functional profiling

**Figure 5:**
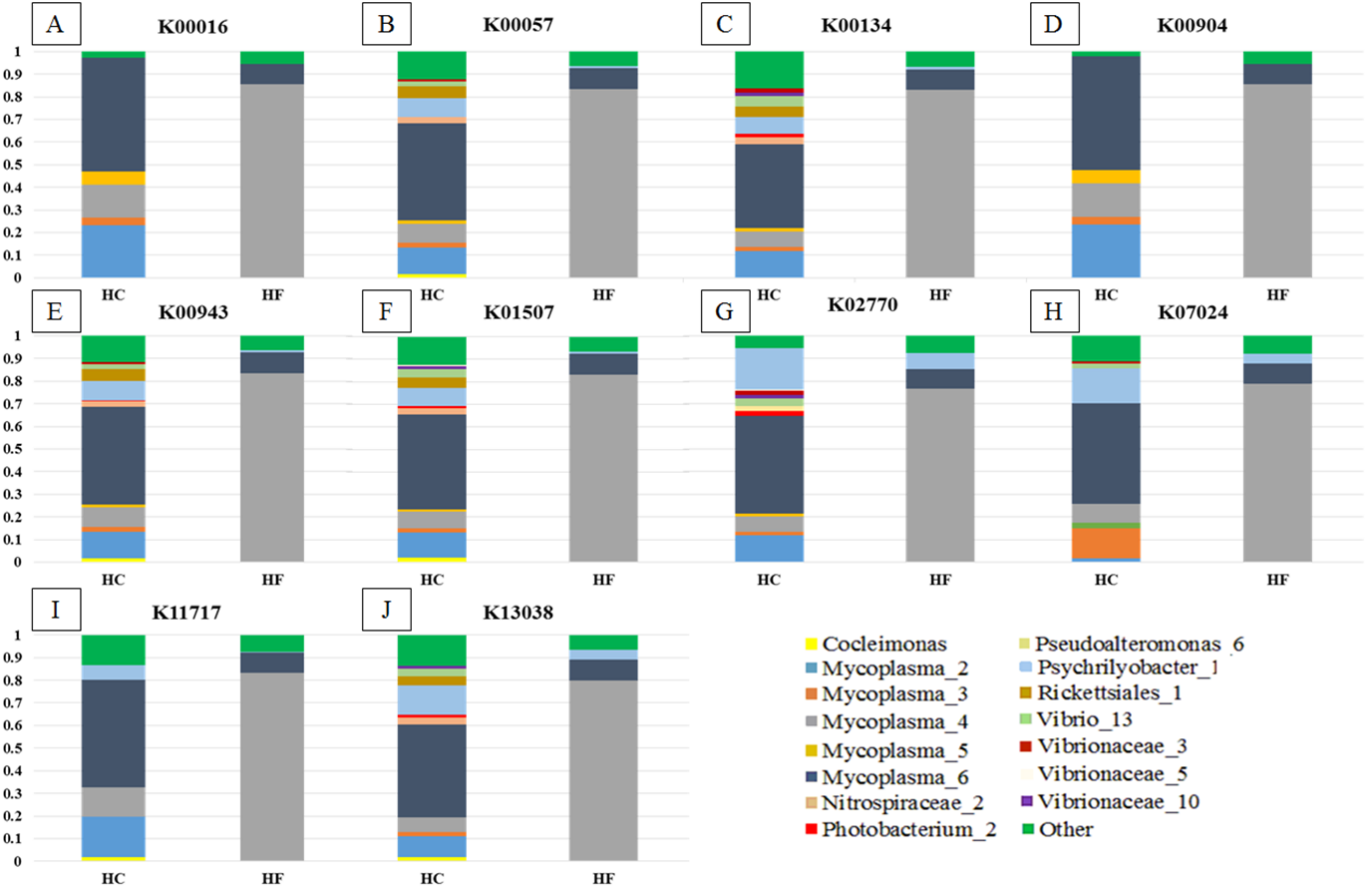
Taxonomic spectra of KEGG orthologs. Bar plots of the contribution of bacterial OTUs (their taxonomic identification color coded in the legend) of ten of the predominant KOs (A to J) chosen at random from the GI microbiomes of *Haliotis fulgens* (HF) and *H. corrugata* (HC). Significant differences between species were found in all KOs ( *χ*2 > 47.74; *p* < 0.001).

Greengenes database, allowed us to detect 248 OTUs, of which 81% (201) were shared with SILVA. Most abalone possessed mean NSTI (Nearest Sequenced Taxon Index) values from 0.07 to 0.17, except for seven HCs with larger values that suggested unreliable functional assignments; hence they were excluded from further analyses. PICRUSt identified 4,092 KOs genes (Table S5) involved in 261 metabolic functions (Table S6). An order of magnitude drop in log-transformed abundance was observed in the ranking of KOs (Fig. S7); hence, *metagenome contributions* analysis was carried out on a random set of 10 of the 86 most abundant KOs (i.e., Log (KOs counts) > 4). According to PICRUSt, the metabolic functions in the HF microbiomes were generally enriched by one primary OTU, whereas many more OTUs contributed to the same function in HC (Fig. 5). The contributions of bacterial OTUs to each KO differed significantly between species (*χ*^2^ >  47.74, *p* < 0.001; Fig. 5). Given their numerical predominance in the metabarcoding reads involved in functional profiling (76%), *Mycoplasma* occupied a major role in the taxonomic spectra of the KOs analyzed (72% in HC, 90% in HF).

**Figure 6:**
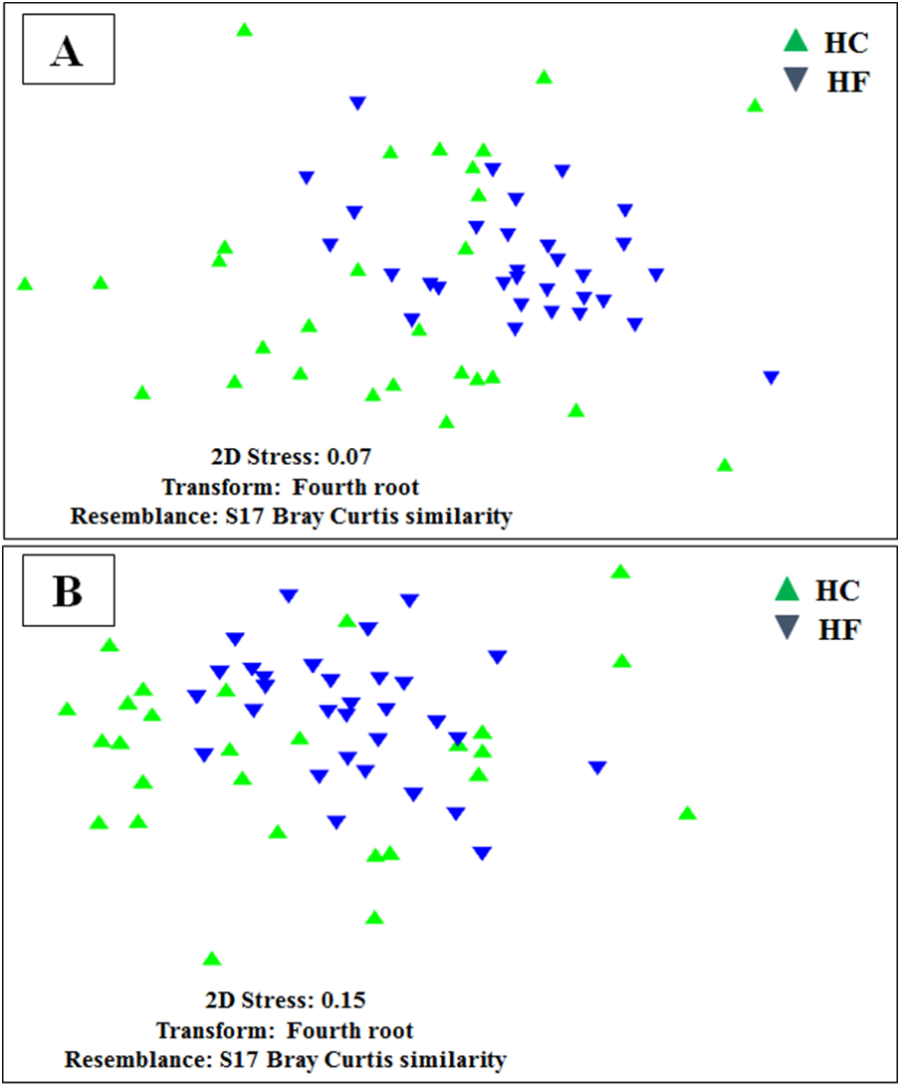
Individual microbiome functional variation. Non-metric multidimensional scaling (MDS) based on Bray Curtis similarity index using fourth root transformed data of: (A) counts of predicted metabolic functions and (B) KEGG orthologue counts in the gut microbiomes of *H. corrugata* (HC) and *Haliotis fulgens* (HF).

MDS analyses performed using both metabolic function counts (Table S5) or KOs counts (Table S6) revealed no clear functional distinction of the GI microbiota of both species. Nevertheless, the scatter of individual microbiomes is much larger in HC, which is consistent with its higher microbial diversity (Fig. 6).

## Discussion

### Abalone microbiome composition

*Mollicutes*, mostly represented by *Mycoplasma* spp., was by far the most abundant class, followed by *Fusobacteria*, *Alphaproteobacteria* and *Gammaproteobacteria*. These bacteria have also been found dominating in the GI microbiota of other abalone species (*H. discus hannai*, *H. diversicolor* and *H. gigantean* (Morales-Bojórquez, Muciño Díaz & Vélez-Barajas, 2008; Tanaka et al., 2003; Macery & Coyne, 2004; Rungrassamee et al., 2014)). Even though the taxonomic composition of HC and HF GI microbiotas bears resemblance at high taxonomic levels, the species level composition showed significant differences. Additionally, unweighted UniFrac suggests that observed differences may reflect microbial evolutionary adaptations to either HC or HF. In this context, several *Mycoplasma* species predominating in one species of abalone were either absent or at low abundance in the other (Table S4). Furthermore, all *Vibrio* spp. possessed a higher prevalence in HC. Following our interpretation, bacterial abundance may be controlled by interspecific competition. Indeed ${\bar {J}}_{d}$ values, suggest that for most bacterial species their abundance increases with decreasing ${\bar {J}}_{d}$, as observed for *Mycoplasma*. Furthermore, ${\bar {J}}_{d}$ for a single bacterium changed according to its host, which suggests that the same bacterial species faces different competitive pressures depending on the microbiota composition. In other words, bacteria that may survive competition in one microbiome, may be out competed in the other.

Some pathogenic bacteria, such as *Candidatus* Xenohaliotis californiensis were observed in the microbiomes of healthy abalone. This bacterium is a pleomorphic, gram-negative coccobacillus that inhabits abalone gastrointestinal epithelia and is considered an obligate endoparasite, like other *Rickettsiales* (Friedman, 2012; Friedman et al., 2000; Moore et al., 2002; Crosson et al., 2014). We observed the presence of *Candidatus* Xenohaliotis californiensis in healthy blue and yellow abalone, which supports that the presence of this pathogen is not sufficient to trigger withering syndrome, as previously suggested (Cáceres-Martínez, Vásquez-Yeomans & Flores-Saaib, 2011). Moreover, its absence and/or low intensity in abalone with morphological and histological signs of withering syndrome has already been reported (Cáceres-Martínez, Vásquez-Yeomans & Flores-Saaib, 2011; Balseiro et al., 2006; Horwitz, Mouton & Coyne, 2016). Accordingly, we posit that further investigations are needed to reveal all the factors involved in withering syndrome outbreaks.

Notably, *Vibrio halioticoli* was absent in both HF and HC whereas it has been found at a prevalence ranging from 40 to 65% in the GI of other marine invertebrates, including several abalone species (Cox, 1962). The discrepancy may in part be related to the anatomical source of the microbiomes, our samples come from post esophageal tissue only whereas other studies have analyzed the entire GI tract (Iehata et al., 2014; Tanaka et al., 2004). Consequently, the presence of *V. halioticoli* in the rest of GI tissues of HC and HF should be explored.

Given that our extraction method does not incorporate a bead-beating step, it may introduce a bias against gram-positive bacteria (Yuan et al., 2012; Gill et al., 2016). However, our gram-specific amplifications revealed that both groups are present in the DNA extracted from the post-esophagus of both species of abalone producing bands of comparable strength in agarose gel electrophoresis. This suggests a lack of bias introduced by the DNA extraction method; however, since endpoint PCR results are not quantitative, they are not conclusive on the extent to which the observed gram-negative bias in the pyrosequenced reads relates to the extraction method. This question requires further attention. Nevertheless, our main findings concern the differences between the sympatric abalone species and do not rely as much on the accuracy of the microbiome estimation, important as it is, but rather on the precision required to differentiate them. Our data proved to be robust in this regard.

### Predicted functions of abalone microbiomes

According to the MDS of KOs gene counts and ecological functions, the inferred functionality of the GI microbiota of HF appears less variable than that of HC, which may reflect a higher degree of diet specialization. The diet diversity of wild HF in Baja California has been shown to be more limited and dominated by *Phyllospadix torreyi* (47%) and algae in the order *Gelidiales* (13%). On the other hand, the diet of sympatric wild HC is more diverse consisting of different species of Phaeophyceae (10–20%), Rhodophyta (20%) and *Gelidiales* (20%) among others (Guzman del Próo, Serviere-Zaragoza & Siqueiros-Beltrones, 2003). These ecological differences are related to their different bathymetric distribution; indeed, HC are generally found in deeper waters (between 10–20 m), whereas HF generally inhabit shallow subtidal regions of rocky shores (between 3–10 m) (Guzman del Próo, Serviere-Zaragoza & Siqueiros-Beltrones, 2003).

PICRUSt analysis, revealed that *Mycoplasma* contributed to all predicted KEGG in both HC and HF. Heretofore, *Mycoplasma* has been considered an obligate parasite and/or commensal due its small genome and limited number of genes (Bano et al., 2007). Nevertheless, according to recent genomic annotation analyses carried out in *Mycoplasma* strains isolated from deep-sea isopods, a mutualistic relationship has been proposed between *Mycoplasma* and their hosts (Wang et al., 2016). Indeed, the genome of studied *Mycoplasma* presented a high number of genes involved in degradation of glycans, proteins and complex oligosaccharides, which supports the hypothesis that *Mycoplasma* may supply their hosts with amino sugars and simple carbohydrates (Wang et al., 2016). Also, given the presence of sialic acid lyase genes, *Mycoplasma* likely protects their hosts against microbial pathogen infections, breaking down the sialic acid cell-wall “coat” used by a great variety of bacterial pathogens to avoid the host’s innate immune response (Wang et al., 2016; Severi, Hood & Thomas, 2007).

Our findings support a mutualistic association between *Mycoplasma* and their abalone hosts; indeed, *Mycoplasma* was the dominant linage in all explored KOs (Fig. 5) which suggests that these bacterial species may play a pivotal role in several metabolic functions. Moreover, a given KO was generally enriched by a single predominant *Mycoplasma* OTU in HF, whereas in HC it was generally enriched by two or more *Mycoplasma* as well as other bacterial-OTUs (Fig. 5). Accordingly, *Mycoplasma* species may be highly host-specific (Register et al., 2015), and they may bear some degree of specificity to particular metabolic functions and/or to specific steps along a metabolic route. Nevertheless, the functional structures of the GI microbiome of both species of abalone bear significant overlap.

## Conclusion

Using bTEFAP we characterized the microbiota of two commercially important and sympatric abalone in Mexico. Our results revealed novel abalone microbiomes with significant shifts in bacterial species composition between them and with other species of abalone in the world. Given that these structural differences in microbiome composition do not necessarily result in distinct functional signatures, we posit that interspecific bacterial competition and the ecological differences of their host (i.e., diet and bathymetric distribution) may be responsible for these differences. These results may provide baseline references for future temporal and spatial sampling, and to assess microbiome changes related to ontogeny as well as physiological/health conditions. Additional efforts should also be directed towards understanding the roles of environment variables or other factors that may alter the GI microbiome of abalone.

##  Supplemental Information

10.7717/peerj.5830/supp-1 Figure S1 Rarefaction analysis for standardization Individual rarefaction curves of the number of observed OTUs against increasing number of individual reads; (A) from HC or yellow and (B) from HF or blue abalone.

10.7717/peerj.5830/supp-2 Figure S2 Unassigned OTUs phylogenetic position Neighbor-joining tree showing the genetic relations between unassigned OTUs reported as **“denovo”** (in back) and other bona fide *16S rRNA* sequences of (GenBank access number in parentheses): bacteria (reported in cardinal red), chloroplasts (reported in green) and Archean species (reported in blue). Numbers at the bifurcations represent bootstrap values calculated on 1,000 pseudo-replicates. Values <70% are not shown.

10.7717/peerj.5830/supp-3 Figure S3 Individual microbiota community structure variation Most abundant bacterial family composition of the post-esophageal microbiota of (A) *H. corrugata* (HC) and (B) *Haliotis fulgens* (HF).

10.7717/peerj.5830/supp-4 Figure S4 Microbiome structural variation using both assigned and unassigned OTUs Non-metric****multidimensional scaling (MDS) based on: (A) Bray Curtis similarity index using fourth root transformed read abundance and (B) Sorensen similarity index based on presence/absence. Both MDSs were obtained using assigned and unassigned OTUs assembled at 97% similarity cut-off of the gut microbiota. HC: *Haliotis corrugata*, HF: *Haliotis fulgens*.

10.7717/peerj.5830/supp-5 Figure S5 Bacterial communities distance Principal coordinate analysis (PCoA) based on unweighted UniFrac distance of bacterial communities harbored by *Haliotis fulgens* and *Haliotis corrugata* abalone, reported as blue and yellow circles respectively.

10.7717/peerj.5830/supp-6 Figure S6 Gram negative and positive-specific amplification results PCR amplification products of the *16S rRNA* gene obtained with primers specific for gram positive or negative bacteria.

10.7717/peerj.5830/supp-7 Figure S7 KOs spectral analysis Ranking of the KO genes according to their count number (log scale) obtained with the script *categorize by function* in PICRUSt.

10.7717/peerj.5830/supp-8 Table S1 Sampling information for each analyzed abalone Columns refer to fishing cooperative name; abalone species ID (HC for yellow abalone or Haliotis corrugata and, HF for blue abalone or Haliotis fulgens) followed by an internal numerical identifier; sampling date for each abalone reported as day/month/year; depth rage of capture (expressed in meters); cooperatives yield processing coordinate.

10.7717/peerj.5830/supp-9 Table S2 OTUs dissimilarity between HF and HC Column abbreviations refers: avarage abundance in HC and HF for each bacterial species (colums C and D respectively), average dissimilarity (column E), dissimilarity percentage of contribution for each bacterial species (column F), cumulative percentage of dissimilarity (column G).

10.7717/peerj.5830/supp-10 Table S3 ID, taxonomy, and read number of each deteced OTUs ID, taxonomy, and read number of OTUs in found yellow (HC) and blue (HF) abalone individuals.

10.7717/peerj.5830/supp-11 Table S4 Differing OTUs between HC and HF OTUs showing differential abundances between yellow (HC, columns from A to D) and blue (HF, columns from F to I) abalone with statistical and biological significance obtained from LEfSe analyses. OTUs are ranked according to their effect size (Log LDA score).

10.7717/peerj.5830/supp-12 Table S5 Functional count number of KEGG Count number in individual abalone and functional description of identified KEGG orthologs.

10.7717/peerj.5830/supp-13 Table S6 Count number of metabolic function Count number of metabolic function genes in individual abalone.

## Additional Information and Declarations

### DNA Deposition

The following information was supplied regarding the deposition of DNA sequences: NCBI Bioproject PRJNA495070.

### Data Availability

The following information was supplied regarding data availability: The raw data are provided in the Supplemental Files.

## References

[ref-1] Backhed F (2005). Host-bacterial mutualism in the human intestine. Science.

[ref-2] Balseiro P, Aranguren R, Gestal C, Novoa B, Figueras A (2006). *Candidatus Xenohaliotis californiensis* and *Haplosporidium montforti* associated with mortalities of abalone *Haliotis tuberculata* cultured in Europe. Aquaculture.

[ref-3] Bano N, DeRae Smith A, Bennett W, Vasquez L, Hollibaugh JT (2007). Dominance of *Mycoplasma* in the guts of the Long-Jawed Mudsucker, *Gillichthys mirabilis*, from five California salt marshes. Environmental Microbiology.

[ref-4] Bates JM, Mittge E, Kuhlman J, Baden KN, Cheesman SE, Guillemin K (2006). Distinct signals from the microbiota promote different aspects of zebrafish gut differentiation. Developmental Biology.

[ref-5] Blaut M, Clavel T (2007). Metabolic diversity of the intestinal microbiota: implications for health and disease. Journal of Nutrition.

[ref-6] Bry L, Falk PG, Midtvedt T, Gordon JI (1996). A model of host-microbial interactions in an open mammalian ecosystem. Science.

[ref-7] Cáceres-Martínez J, Vásquez-Yeomans R, Flores-Saaib RD (2011). Intracellular prokaryote *Xenohaliotis californiensis* in abalone *Haliotis* spp. from Baja California, México. Ciencia Pesquera.

[ref-8] Caporaso JG, Bittinger K, Bushman FD, Desantis TZ, Andersen GL, Knight R (2010a). PyNAST: a flexible tool for aligning sequences to a template alignment. Bioinformatics.

[ref-9] Caporaso JG, Kuczynski J, Stombaugh J, Bittinger K, Bushman FD, Costello EK, Fierer N, Gonzalez AP, Goodrich JK, Gordon JI, Huttley GA, Kelley ST, Knights D, Koenig JE, Ley RE, Lozupone CA, McDonald D, Muegge BD, Pirrung M, Reeder J, Sevinsky JR, Turnbaugh PJ, Walters WA, Widmann J, Yatsunenko T, Zaneveld J, Knight R (2010b). QIIME allows analysis of high-throughput community sequencing data. Nature Methods.

[ref-10] Chao A (1984). Nonparametric estimation of the number of classes in a population. Scandinavian Journal of Statistics.

[ref-11] Clarke KR, Warwick RM (2001). A further biodiversity index applicable to species lists: variation in taxonomic distinctness. Marine Ecology Progress Series.

[ref-12] Colwell RK (2013). http://purl.oclc.org/estimates.

[ref-13] Cox KW (1962). California Abalones, Family Haliotidae. The resources ageny of California department of fish and game. Fishery Bulletin.

[ref-14] Crosson LM, Friedman CS (2017). Withering syndrome susceptibility of northeastern pacific abalones: a complex relationship with phylogeny and thermal experience. Journal of Invertebrate Pathology.

[ref-15] Crosson LM, Wight N, VanBlaricom GR, Kiryu I, Moore JD, Friedman CS (2014). Abalone withering syndrome: distribution, impacts, current diagnostic methods and new findings. Diseases of Aquatic Organisms.

[ref-16] De Voogd NJ, Cleary DFR, Polónia ARM, Gomes NCM (2015). Bacterial community composition and predicted functional ecology of sponges, sediment and seawater from the thousand islands reef complex, West Java, Indonesia. FEMS Microbiology Ecology.

[ref-17] Dowd SE, Callaway TR, Wolcott RD, Sun Y, McKeehan T, Hagevoort RG, Edrington TS (2008). Evaluation of the bacterial diversity in the feces of cattle using 16S rDNA bacterial tag-encoded FLX amplicon pyrosequencing (bTEFAP). BMC Microbiology.

[ref-18] Edgar RC (2010). Search and clustering orders of magnitude faster than BLAST. Bioinformatics.

[ref-19] Friedman CS (2012). Infection with *Xenohaliotis californiensis*. IE- manual of diagnostic test for aquatic animals.

[ref-20] Friedman CS, Andree KB, Beauchamp KA, Moore JD, Robbins TT, Shields JD, Hedrick RP (2000). “*Candidatus* Xenohaliotis californiensis”, a newly described pathogen of abalone, *Haliotis* spp. along the west coast of North America. International Journal of Systematic and Evolutionary Microbiology.

[ref-21] Gill C, Van de Wijgert JHHM, Blow F, Darby AC (2016). Evaluation of lysis methods for the extraction of bacterial DNA for analysis of the vaginal microbiota. PLOS ONE.

[ref-22] Guzman del Próo S, Serviere-Zaragoza E, Siqueiros-Beltrones D (2003). Natural diet of juvenile abalone *Haliotis fulgens* and *H. corrugata* (Mollusca: Gastropoda) in Bahia Tortugas, Mexico. Pacific Science.

[ref-23] Hammer Ø, Harper DAT, Ryan PD (2001). Paleontological statistics software package for education and data analysis. Palaeontologia Electronica.

[ref-24] Horwitz R, Mouton A, Coyne VE (2016). Characterization of an intracellular bacterium infecting the digestive gland of the South African abalone Haliotis midae. Aquaculture.

[ref-25] Huang Z-B, Guo F, Zhao J, Li W-D, Ke C-H (2010). Molecular analysis of the intestinal bacterial flora in cage-cultured adult small abalone, *Haliotis diversicolor*. Aquaculture Research.

[ref-26] Iehata S, Nakano M, Tanaka R, Maeda H (2014). Modulation of gut microbiota associated with abalone *Haliotis gigantea* by dietary administration of host-derived *Pediococcus* sp. Ab1. Fisheries Science.

[ref-27] Klausegger A, Hell M, Berger A, Zinober K, Baier S, Jones N, Wolfgang S, Barbara K (1999). Gram type-specific broad-range PCR amplification for rapid detection of 62 pathogenic bacteria. Journal of Clinical Microbiology.

[ref-28] Langille M, Zaneveld J, Caporaso JG, McDonald D, Knights D, Reyes JA, Clemente JC, Burkepile DE, Thurber RL, Knight R, Beiko RG, Huttenhower C (2013). Predictive functional profiling of microbial communities using 16S rRNA marker gene sequences. Nature Biotechnology.

[ref-29] Lozupone CA, Hamady M, Kelley ST, Knight R (2007). Quantitative and qualitative beta diversity measures lead to different insights into factors that structure microbial communities. Applied and Environmental Microbiology.

[ref-30] Lozupone C, Knight R (2005). UniFrac: a new phylogenetic method for comparing microbial communities unifrac: a new phylogenetic method for comparing microbial communities. Applied and Environmental Microbiology.

[ref-31] Ludwig W, Mittenhuber G, Friedrich CG (1993). Transfer of *Thiosphaera pantotropha* to *Paracoccus denitrificans*. International Journal of Systematic Bacteriology.

[ref-32] Macery B, Coyne VE (2004). Improved growth rate and disease resistance in farmed *Haliotis midae* through probiotic treatment. Aquaculture.

[ref-33] Meryandini A, Junior MZ, Rusmana I (2015). The role of agarolytic bacteria in enhancing physiological function for digestive system of abalone (*Haliotis asinina*). Journal of Applied Environmental and Biological Sciences.

[ref-34] Moore JD, Finley CA, Friedman CS, Robbins TT (2002). Withering syndrome and restoration of southern California abalone populations. California Cooperative Oceanic Fisheries Investigations Report.

[ref-35] Morales-Bojórquez E, Muciño Díaz MO, Vélez-Barajas JA (2008). Analysis of the decline of the abalone fishery (*Haliotis fulgens* and *H. corrugata*) along the Westcentral Coast of the Baja California Peninsula, Mexico. Journal of Shellfish Research.

[ref-36] Pang SJ, Xiao T, Bao Y (2006). Dynamic changes of total bacteria and *Vibrio* in an integrated seaweed–abalone culture system. Aquaculture.

[ref-37] Rahel FJ (2000). Homogenization of fish faunas across the United States. Science.

[ref-38] Register KB, Thole L, Rosenbush RF, Minion FC (2015). Multilocus sequence typing of *Mycoplasma bovis* reveals host-specific genotypes in cattle versus bison. Veterinary Microbiology.

[ref-39] Ruff-Roberts AL, Kuenen JG, Ward DM (1994). Distribution of cultivated and uncultivated cyanobacteria and *Chloroflexus*-like bacteria in hot spring microbial mats. Applied and Environmental Microbiology.

[ref-40] Rungrassamee W, Klanchui A, Maibunkaew S, Chaiyapechara S, Jiravanichpaisal P, Karoonuthaisiri N (2014). Characterization of intestinal bacteria in wild and domesticated adult black tiger shrimp (*Penaeus monodon*). PLOS ONE.

[ref-41] SAGARPA (2009). Sustentabilidad y Pesca Responsable en México. Evaluación y Manejo.

[ref-42] Sawabe T, Setoguchi N, Inoue S, Tanaka R, Ootsubo M, Yoshimizu M, Ezura Y (2003). Acetic acid production of *Vibrio halioticoli* from alginate: a possible role for establishment of abalone *V. halioticoli* association. Aquaculture.

[ref-43] Segata N, Izard J, Waldron L, Gevers D, Miropolsky L, Garrett WS, Huttenhower C (2011). Metagenomic biomarker discovery and explanation. Genome Biology.

[ref-44] Severi E, Hood DW, Thomas GH (2007). Sialic acid utilization by bacterial pathogens. Microbiology.

[ref-45] Tamura K, Stecher G, Peterson D, Filipski A, Kumar S (2013). MEGA6: molecular evolutionary genetics analysis version 6. Molecular Biology and Evolution.

[ref-46] Tanaka R, Ootsubo M, Sawabe T, Ezura Y, Tajima K (2004). Biodiversity and in situ abundance of gut microflora of abalone (*Haliotis discus hannai*) determined by culture-independent techniques. Aquaculture.

[ref-47] Tanaka R, Sugimura I, Sawabe T, Yoshimizu M, Ezura Y (2003). Gut microflora of abalone *Haliotis discus hannai*. Fisheries Science.

[ref-48] Ten Doeschate KI, Coyne VE (2008). Improved growth rate in farmed *Haliotis midae* through probiotic treatment. Aquaculture.

[ref-49] Urbanová Z, Bárta J (2014). Microbial community composition and in silico predicted metabolic potential reflect biogeochemical gradients between distinct peatland types. FEMS Microbiology Ecology.

[ref-50] Vázquez-Baeza Y, Pirrung M, Gonzalez A, Knight R (2013). EMPeror: a tool for visualizing high-throughput microbial community data. Gigascience.

[ref-51] Wang Y, Huang JM, Wang SL, Gao ZM, Zhang AQ, Danchin A, He LS (2016). Genomic characterization of symbiotic mycoplasmas from the stomach of deep-sea isopod bathynomus sp. Environmental Microbiology.

[ref-52] Weiss S, Xu ZZ, Peddada S, Amir A, Bittinger K, Gonzalez A, Lozupone C, Zaneveld JR, Vázquez-Baeza Y, Birmingham A, Hyde ER, Knight R (2017). Normalization and microbial differential abundance strategies depend upon data characteristics. Microbiome.

[ref-53] Yuan S, Cohen DB, Ravel J, Abdo Z, Forney LJ (2012). Evaluation of Methods for the Extraction and Purification of DNA from the Human Microbiome. PLOS ONE.

[ref-54] Zhao J, Shi B, Jiang QR, Ke CH (2012). Changes in gut-associated flora and bacterial digestive enzymes during the development stages of abalone (*Haliotis diversicolor*). Aquaculture.

