# Supplementary figures and images for: Structure, dynamics and predicted functional role of the gut microbiota of the blue (Haliotis fulgens) and yellow (H. corrugata) abalone from Baja California Sur, Mexico
Source: PeerJ. 2018 Nov 2;6:e5830. doi: 10.7717/peerj.5830 (PMC6216945; doi:10.7717/peerj.5830)

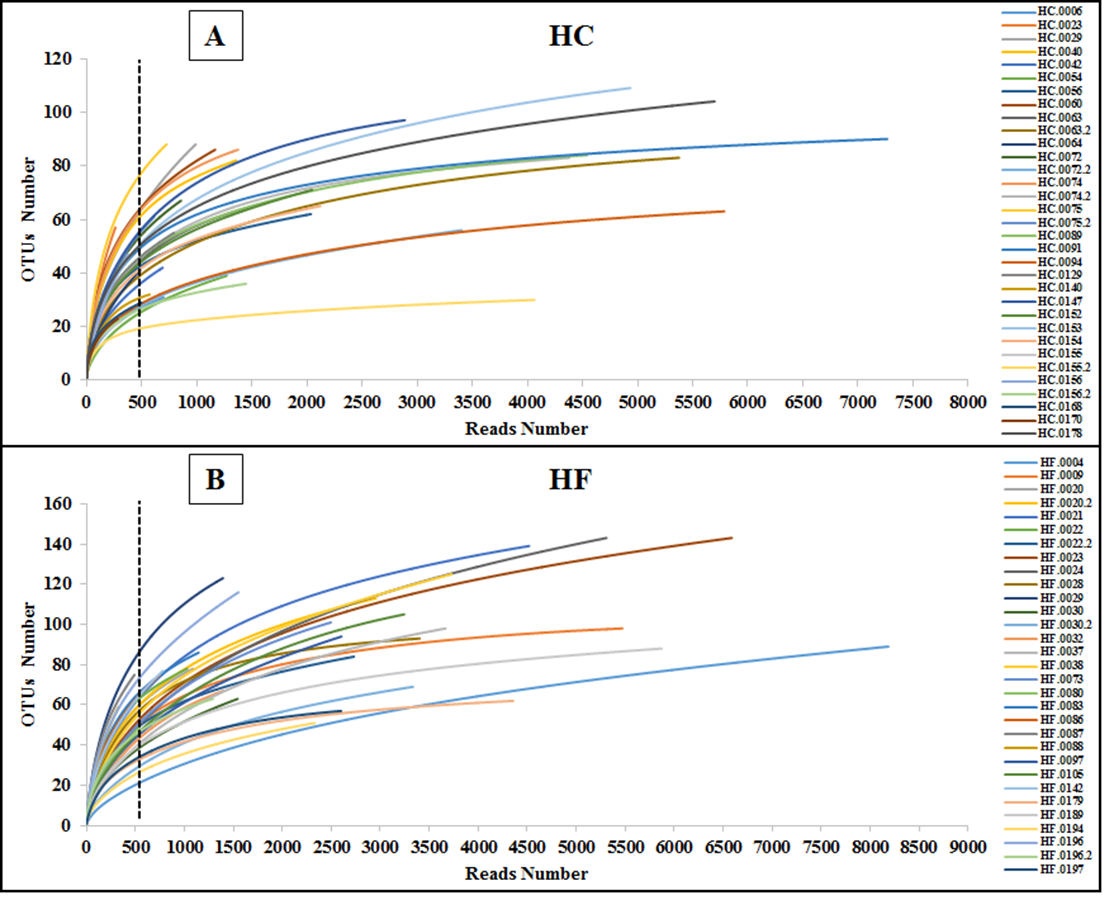

Supplement: Figure S1 — Individual rarefaction curves of the number of observed OTUs against increasing number of individual reads; (A) from HC or yellow and (B) from HF or blue abalone. [file peerj-06-5830-s001.png]

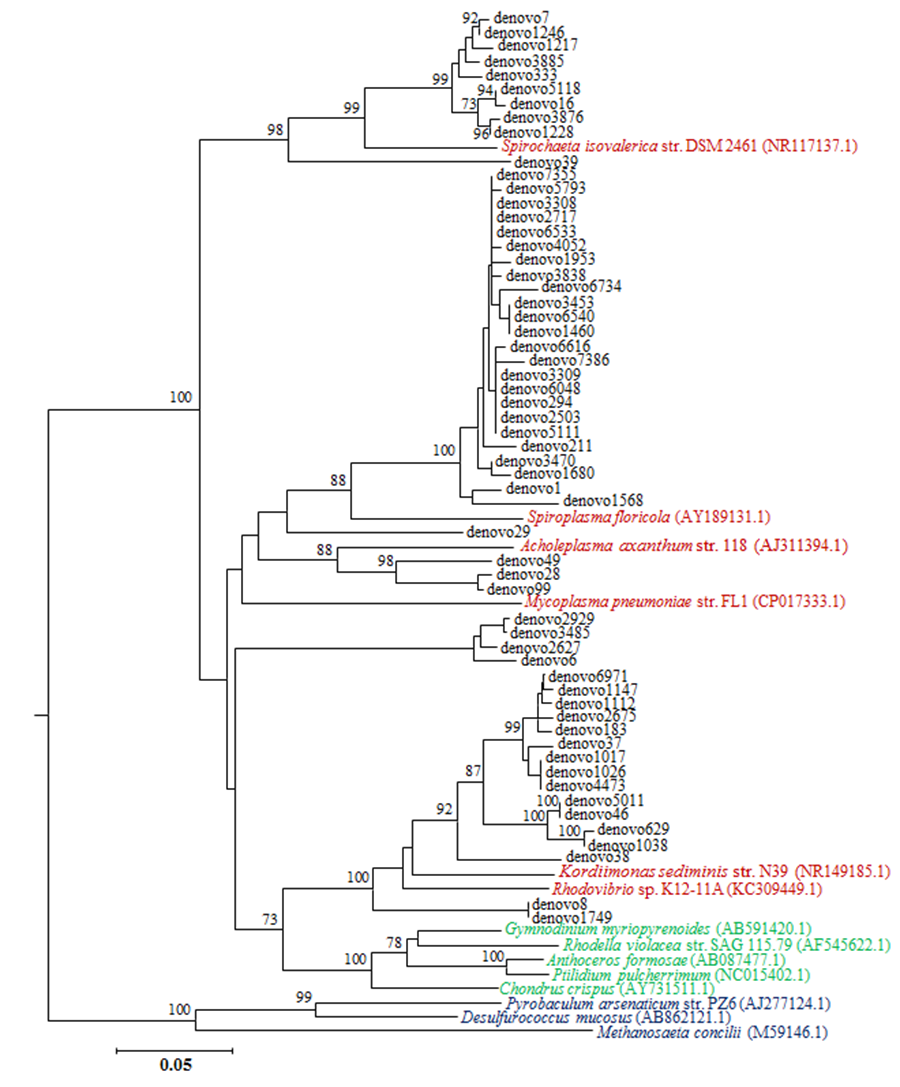

Supplement: Figure S2 — Neighbor-joining tree showing the genetic relations between unassigned OTUs reported as “denovo” (in back) and other bona fide 16S rRNA sequences of (GenBank access number in parentheses): bacteria (reported in cardinal red), chloroplasts (reported in green) and Archean species (reported in blue). Numbers at the bifurcations represent bootstrap values calculated on 1,000 pseudo-replicates. Values <70% are not shown. [file peerj-06-5830-s002.png]

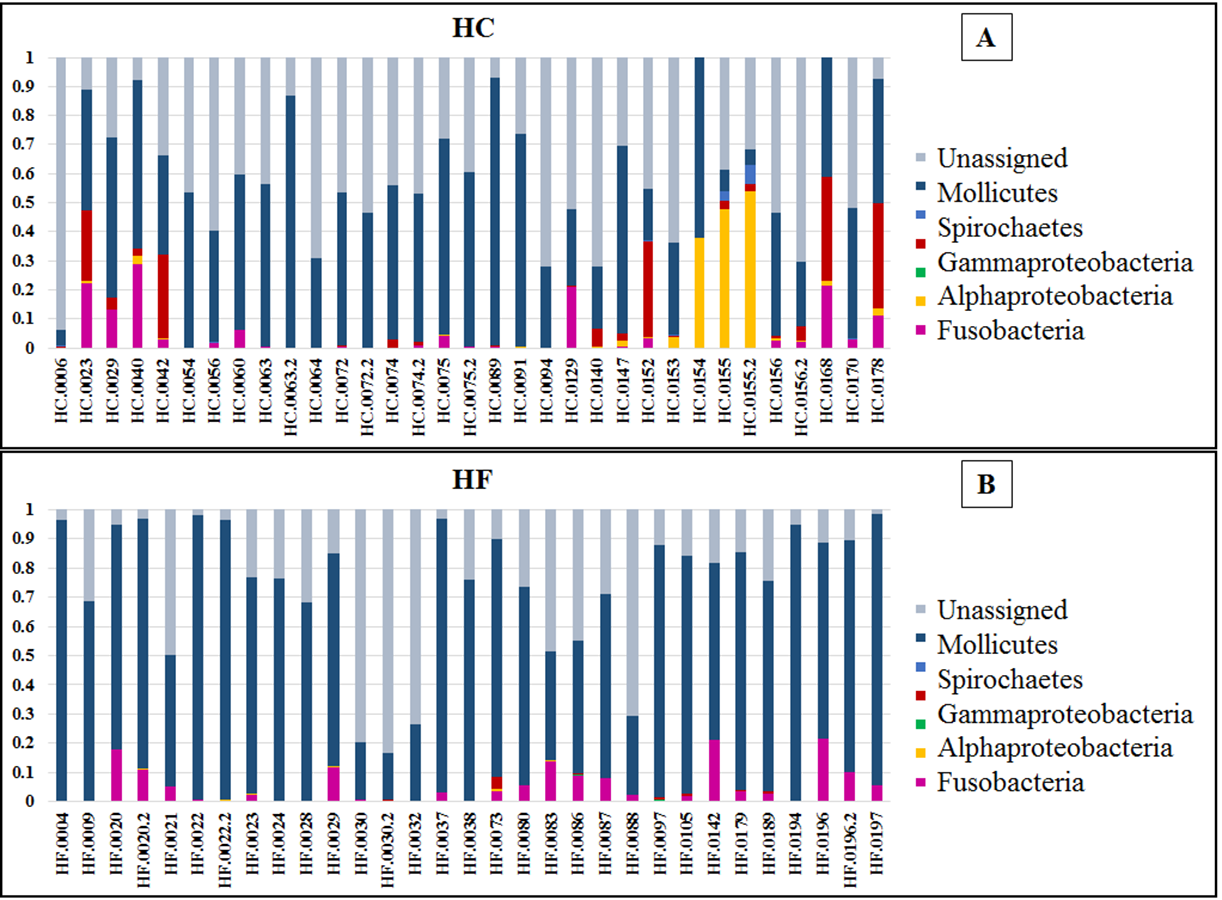

Supplement: Figure S3 — Most abundant bacterial family composition of the post-esophageal microbiota of (A) H. corrugata (HC) and (B) Haliotis fulgens (HF). [file peerj-06-5830-s003.png]

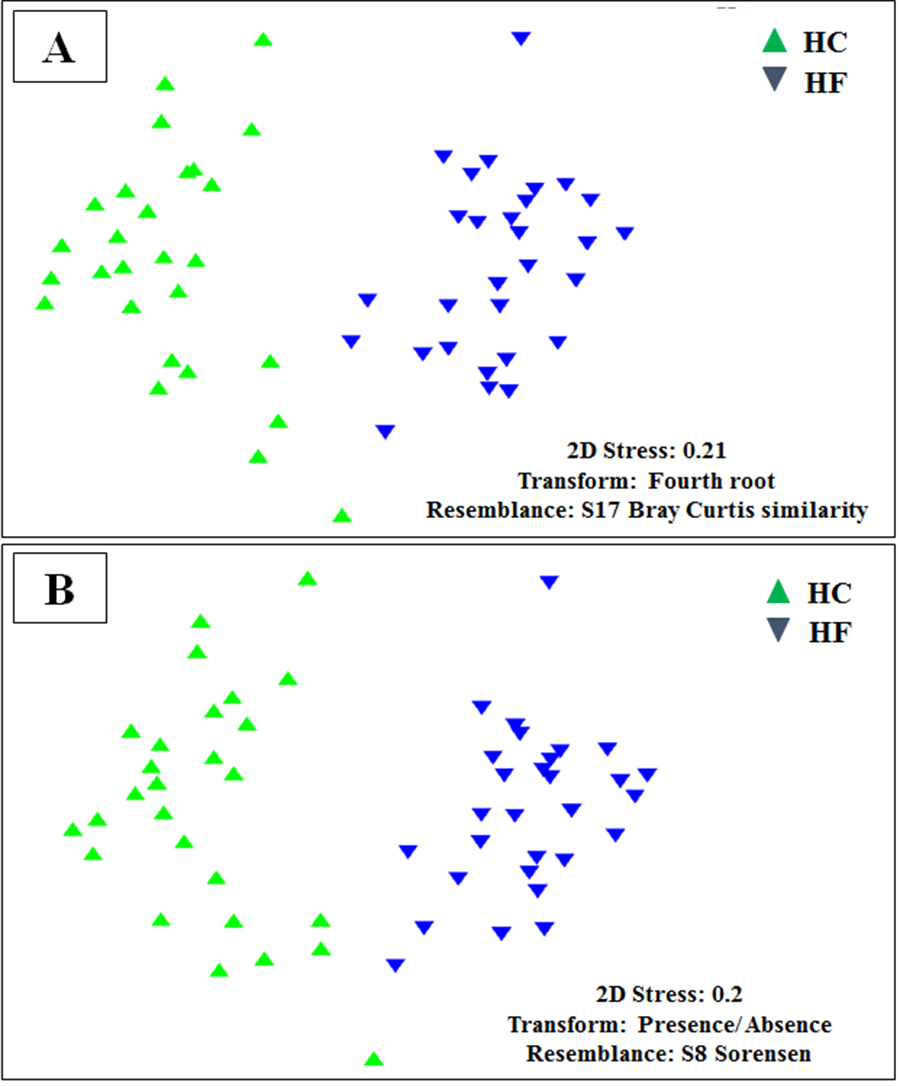

Supplement: Figure S4 — Non-metricmultidimensional scaling (MDS) based on: (A) Bray Curtis similarity index using fourth root transformed read abundance and (B) Sorensen similarity index based on presence/absence. Both MDSs were obtained using assigned and unassigned OTUs assembled at 97% similarity cut-off of the gut microbiota. HC: Haliotis corrugata, HF: Haliotis fulgens. [file peerj-06-5830-s004.png]

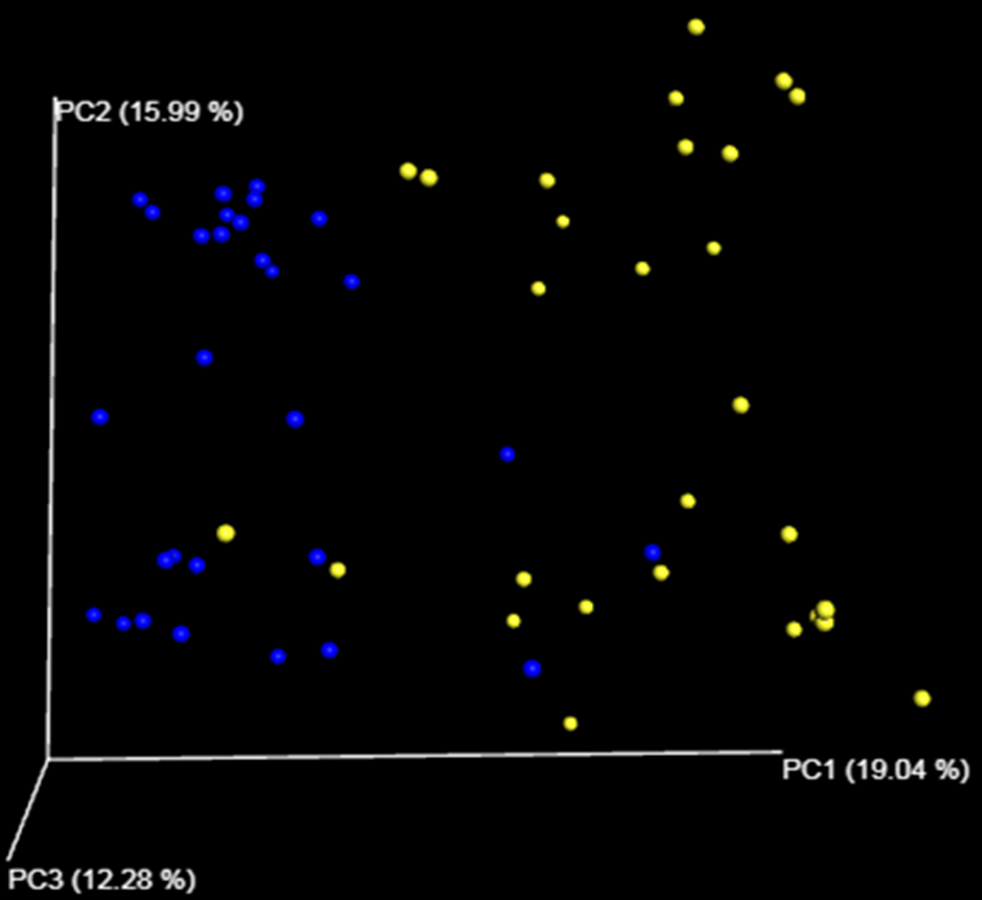

Supplement: Figure S5 — Principal coordinate analysis (PCoA) based on unweighted UniFrac distance of bacterial communities harbored by Haliotis fulgens and Haliotis corrugata abalone, reported as blue and yellow circles respectively. [file peerj-06-5830-s005.png]

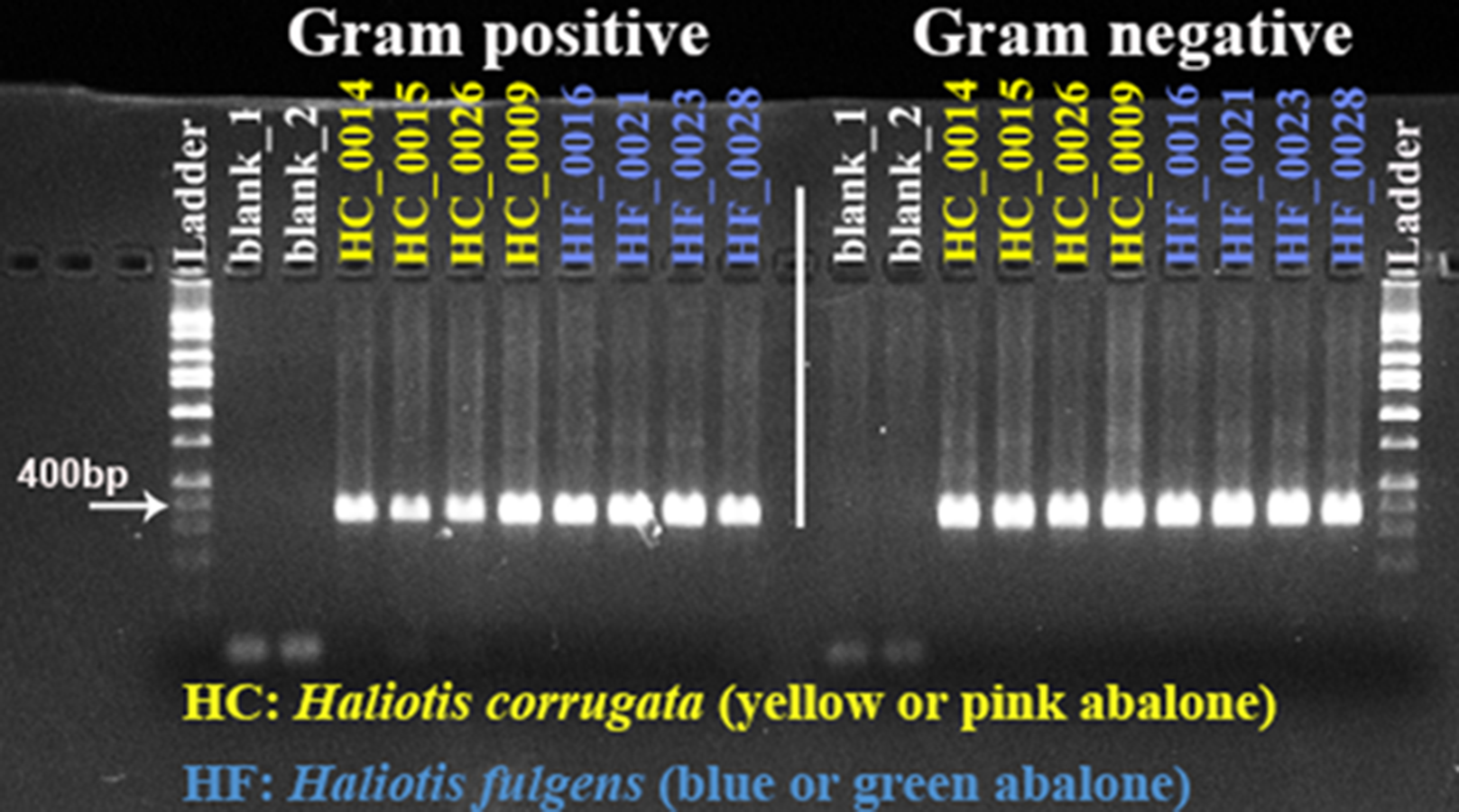

Supplement: Figure S6 — PCR amplification products of the 16S rRNA gene obtained with primers specific for gram positive or negative bacteria. [file peerj-06-5830-s006.png]

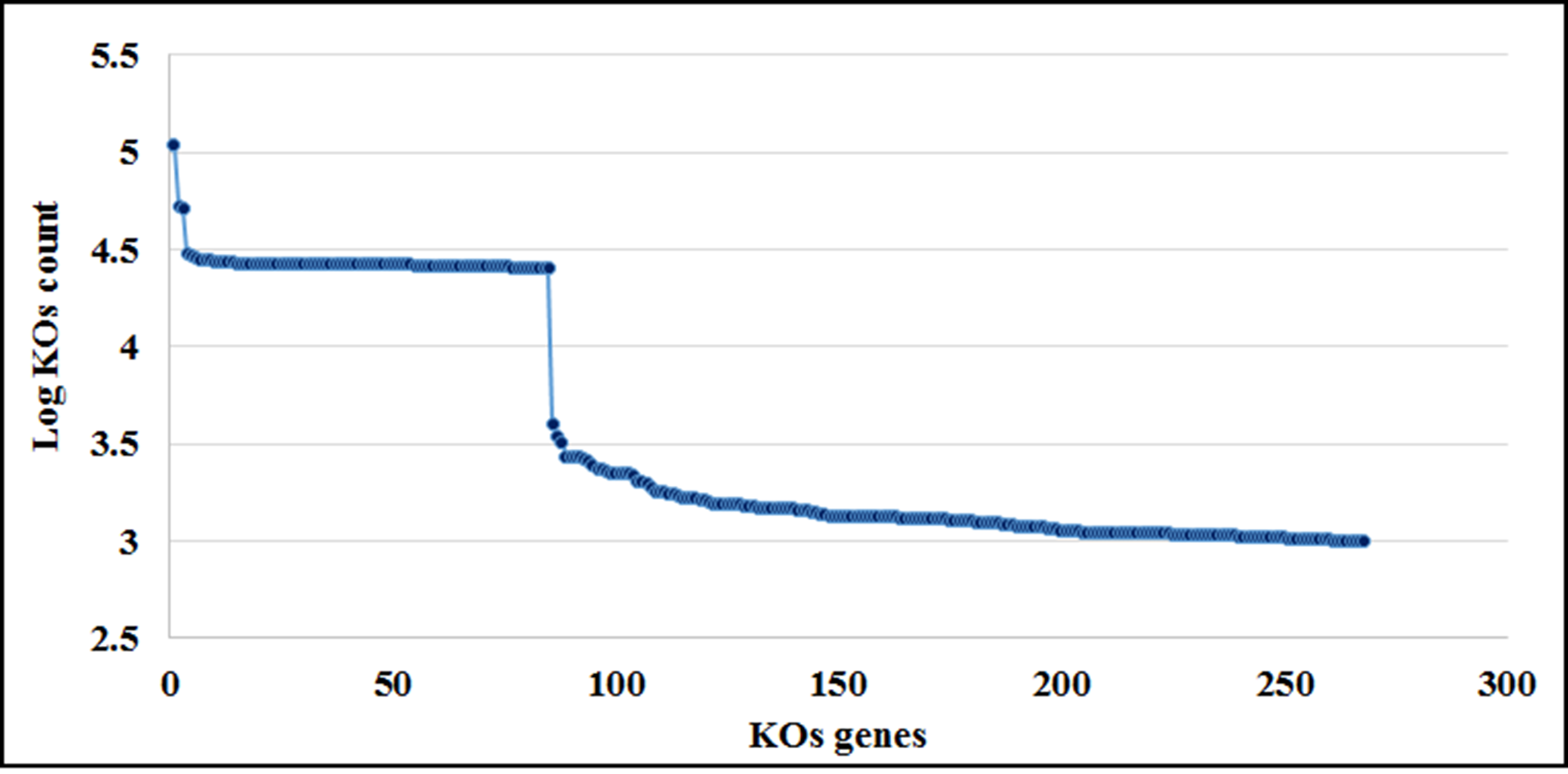

Supplement: Figure S7 — Ranking of the KO genes according to their count number (log scale) obtained with the script categorize by function in PICRUSt. [file peerj-06-5830-s007.png]
